# A Digital Health Intervention for Stress and Anxiety Relief in Perioperative Care: Protocol for a Feasibility Randomized Controlled Trial

**DOI:** 10.2196/38536

**Published:** 2022-11-29

**Authors:** Haridimos Kondylakis, Irene Alice Chicchi Giglioli, Dimitrios G Katehakis, Hatice Aldemir, Paul Zikas, George Papagiannakis, Santiago Hors-Fraile, Pedro L González-Sanz, Konstantinos C Apostolakis, Constantine Stephanidis, Francisco J Núñez-Benjumea, Rosa M Baños-Rivera, Luis Fernandez-Luque, Angelina Kouroubali

**Affiliations:** 1 Institute of Computer Science, Foundation for Research and Technology (FORTH) Heraklion Greece; 2 Adhera Health, Inc Palo Alto, CA United States; 3 Digital Health Program Barcelona Technology School Barcelona University Barcelona Spain; 4 ORamaVR SA Heraklion Greece; 5 Department of Computer Science, University of Crete Heraklion Greece; 6 Virgen Macarena University Hospital, Andalusian Health Service Sevilla Spain; 7 Polibienestar Institute, University of Valencia Valencia Spain; 8 CiberObn Pathophysiology of Obesity and Nutrition (CB06/03), Instituto Salud Carlos III Madrid Spain

**Keywords:** CARINAE, digital health, perioperative process, patient empowerment, stress and anxiety management, mobile health, mHealth, virtual reality, VR, health recommender system, HRS

## Abstract

**Background:**

Stress and anxiety are psychophysiological responses commonly experienced by patients during the perioperative process that can increase presurgical and postsurgical complications to a comprehensive and positive recovery. Preventing and intervening in stress and anxiety can help patients achieve positive health and well-being outcomes. Similarly, the provision of education about surgery can be a crucial component and is inversely correlated with preoperative anxiety levels. However, few patients receive stress and anxiety relief support before surgery, and resource constraints make face-to-face education sessions untenable. Digital health interventions can be helpful in empowering patients and enhancing a more positive experience. Digital health interventions have been shown to help patients feel informed about the possible benefits and risks of available treatment options. However, they currently focus only on providing informative content, neglecting the importance of personalization and patient empowerment.

**Objective:**

This study aimed to explore the feasibility of a digital health intervention called the Adhera CARINAE Digital Health Program, designed to provide evidence-based, personalized stress- and anxiety-management methods enabled by a comprehensive digital ecosystem that incorporates wearable, mobile, and virtual reality technologies. The intervention program includes the use of advanced data-driven techniques for tailored patient education and lifestyle support.

**Methods:**

The trial will include 5 hospitals across 3 European countries and will use a randomized controlled design including 30 intervention participants and 30 control group participants. The involved surgeries are cardiopulmonary and coronary artery bypass surgeries, cardiac valve replacement, prostate or bladder cancer surgeries, hip and knee replacement, maxillofacial surgery, or scoliosis. The control group will receive standard care, and the intervention group will additionally be exposed to the digital health intervention program.

**Results:**

The recruitment process started in January 2022 and has been completed. The primary impact analysis is currently ongoing. The expected results will be published in early 2023.

**Conclusions:**

This manuscript details a comprehensive protocol for a study that will provide valuable information about the intervention program, such as the measurement of comparative intervention effects on stress; anxiety and pain management; and usability by patients, caregivers, and health care professionals. This will contribute to the evidence planning process for the future adoption of diverse digital health solutions in the field of surgery.

**Trial Registration:**

ClinicalTrials.gov NCT05184725; https://www.clinicaltrials.gov/ct2/show/NCT05184725

**International Registered Report Identifier (IRRID):**

DERR1-10.2196/38536

## Introduction

### Background

Patients undergoing surgical operations exhibit symptoms of severe stress, anxiety, and fear as common physiological and psychological reactions to potentially threatening situations associated with surgeries [1,2]. Family caregivers are expected to assume a complex caregiving role for these patients and are therefore confronted with emotional distress and physical decline as well [3].

Surgeons and health care professionals (HCPs) use a variety of stress-coping strategies, as stressors that affect surgical performance and contribute to complications [4]. Psychological support and patient education have proven to be very effective in reducing this stress and anxiety [5,6]. Provision of information about their surgery constitutes an essential part of the preoperative experience both in patients and their caregivers because it helps decrease anxiety levels as well as surgical complications [7,8]. Patient empowerment improves self-care management, encourages patients to take an active role in managing their diseases, and expands outcomes such as satisfaction, cost, health status, and function [9]. Addressing caregiver strain is also an important aspect of care for pediatric patients [10] and those with special health care needs [11].

Advances in digital health interventions have been a great support for enhancing awareness about health conditions and for the management of mental health by relying on both nonimmersive systems (eg, mobile apps) and immersive systems (eg, virtual reality [VR]) [12]. These include aspects such as supporting patients in the management of anxiety, stress [13], and pain [14,15]. VR has been used in multiple health care applications, including reducing stress and pain, training medical practitioners, patient counseling, cognitive rehabilitation, physical therapy in medicine, diagnostic and treatment needs in dentistry, in mental health management, and surgery [16]. A multiuser immersive VR (IVR) system was developed and used during presurgical discussions in a prospective patient cohort undergoing cerebrovascular surgery [17]. An IVR intervention adopted in pediatric patients to manage pain and anxiety provides a new, easy, and cost-effective intervention that can be applied to other painful and stressful medical procedures [18]. Pain is a highly distressing symptom for patients in all clinical settings, and it is influenced by stress and anxiety levels. VR applications have proven to be efficient in stress relief and pain management mainly because of their distractive properties [19].

Digital health interventions generate substantial amounts of data that can be used for personalization, relying on artificial intelligence (AI) techniques [20-24], including applications in perioperative care users [25]. A particular type of AI-based system is the health recommender systems (HRSs), which enable the personalization of patient interventions based on their unique behavioral and health needs [26,27]. For example, perioperative stress is perceived very differently from patient to patient and varies largely with the severity of the illness and the required surgery type. Therefore, the effort to assess stress levels and interventions to develop the skills needed to better cope with it should be a personalized experience related to health behavior theories. HRSs incorporate behavioral change models such as I-Change to guide the personalization of educational and behavioral intervention [28,29].

There is a lack of understanding of the feasibility of combining VR with mobile-based technologies as the main channel for the provision of digital health intervention [30]. In this study, what is explored is the feasibility of a digital health intervention that leverages latest mobile and VR technologies within the use case of helping patients to manage stress and anxiety during surgery while promoting healthy recovery.

On the basis of these premises, the aim of this study was to explore the trial design and effect of a new digital health intervention called the Adhera CARINAE Digital Program that combines evidence-based perioperative stress management, anxiety, and pain relief techniques grounded in behavioral science with relevant education and information resources. As such, this study aimed to explore whether using an advanced digital health platform, which provides evidence-based, personalized stress- and anxiety-management methods, through the designed intervention can reduce perioperative stress. The results of the intervention were measured through a series of self-reported measurements and compared with versus of a control group to determine the effect of the intervention.

### The Adhera CARINAE Digital Health Program Overview

Digital health interventions designed to help patients before, during, and after surgery reduce their stress and anxiety levels and improve their overall self-management, including support for positive lifestyle changes [29]. Within the context of this study, the selected clinical use cases were cardiac, traumatological, and oncology surgeries, including both pediatric and adult patients. In the case of pediatrics, the key users include caregivers.

The development of digital intervention has followed an iterative, detailed requirement elicitation process involving end users and capitalizing many years of work on personal health systems [31-34]. It combines various multichannel technologies as well as evidence-based content to support patients (including children and older adults) in their perioperative journey, as well as caregivers and HCP. The targeted surgeries include, but are not limited to, cardiopulmonary and coronary artery bypass surgery, cardiac valve replacement, prostate, kidney, and bladder cancer surgery, hip and knee replacement, maxillofacial surgery, orthognathic surgery, and scoliosis surgery. The design of the intervention was based on input from (1) personalized recommendations and educational content, (2) communication with HCPs, and (3) activities on mental well-being [26,35]. All modules were designed to be flexible and adaptable to accommodate national, regional, local, and institutional policies and guidelines. The embedded educational content is based on clinical guidelines and approved by clinicians, whereas the content has been generated by a multidisciplinary team including HCPs, surgeons, and psychologists. Interoperability is a key advantage of the system, as it can export or import data using both existing standards and proprietary mechanisms.

**Figure 1:**
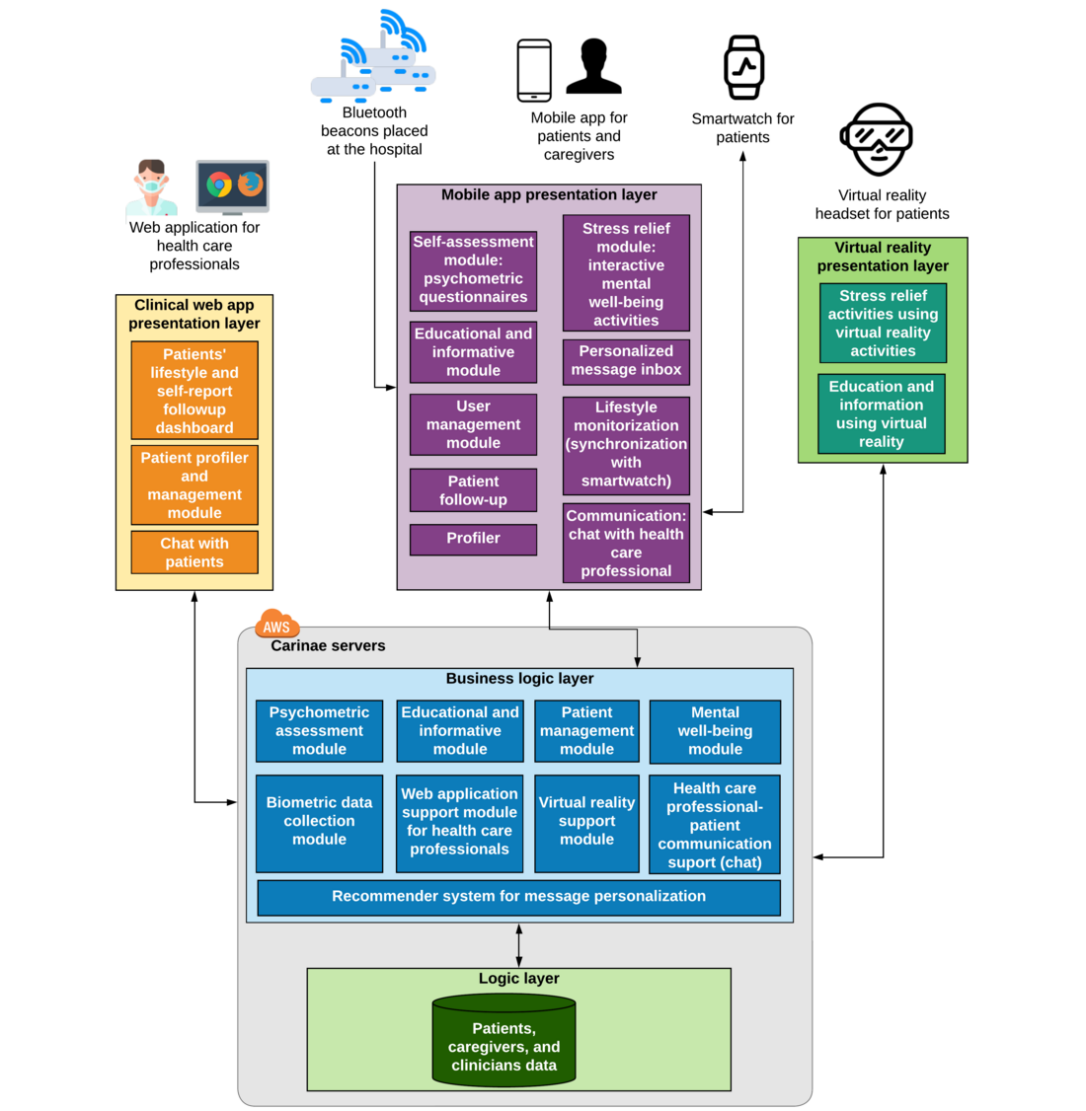
Architecture overview and functionalities embedded into the Adhera CARINAE Digital Program intervention. AWS: Amazon Web Services.

The digital intervention program delivers nonmedical intervention to patients, including (1) personalized health education to improve patient self-management skills addressing surgery needs and recovery, (2) behavioral motivational messages aimed at promoting healthier lifestyles, (3) mental well-being support to reduce stress and anxiety, and (4) a collaborative platform to enable collaboration between patients, caregivers, and HCPs (Figure 1). The program is delivered using a digital health ecosystem that leverages the following key elements:

- Mobile app: This incorporates AI-based behavioral coaching based on the Adhera Health Precision Digital Companion Platform that incorporates an advanced HRS, which is complemented by educational content and mental well-being exercises based on cognitive behavioral therapy. The mobile solution also integrates wearable technologies (a Withings Pulse HR smartwatch) and Internet of Things (IoT) technology (location beacons in the hospital) to support patient monitoring and personalization.
- VR component: This provides a combination of educational content and mental well-being exercises.
- Clinical web application: This provides patient support dashboards and tools for HCPs involved in patient care (eg, chat and monitoring).

Patient information required for digital intervention includes minimal demographic information, patient medical records, and preferences and is securely stored in European Amazon Servers, respecting all national, international, and European regulations and aligned with ISO 27001. Data are encrypted, both in transit and at rest, and only minimal personal information required is stored.

### The Digital Health Program: Mobile App Component

**Figure 2:**
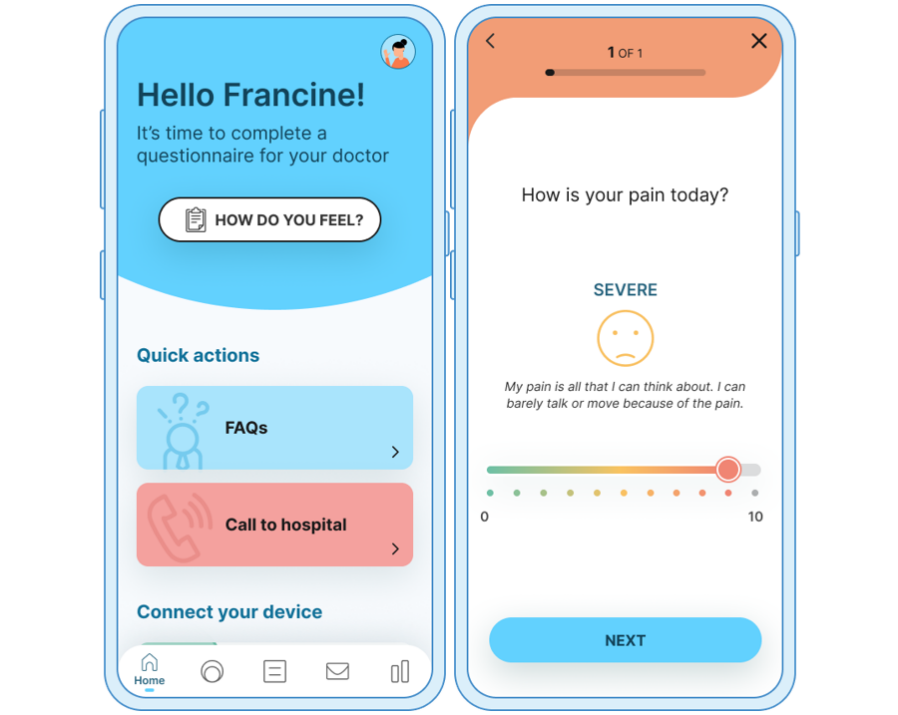
The Adhera Health CARINAE Digital Program mobile app.

The intervention mobile app (iOS and Android app) was designed to support patients before, during, and after surgery with a set of features (Figure 2). The underlying principle of the mobile component is to support (1) patient self-management education, (2) stress reduction techniques via mobile-based exercises, (3) behavioral motivation, and (4) collaboration between clinicians and caregivers.

The educational and information modules of the mobile solution are designed to support self-management skills and related knowledge using principles set up by the Patient Education Materials Assessment Tool recommendations regarding understandability and actionability [33]. These modules leverage the patients’ profile details to present a set of relevant educational contents about their surgery as well as beneficial information to prepare patients physically and emotionally. These contents were complemented with related quiz questions to maximize knowledge retention. The psychoeducational and information modules support patients throughout the 3 main stages of the patient journey: (1) thirteen educational modules aimed at preparing for the surgery (eg, knowledge about the surgery, coping mechanism for fear and stress, self-care for preparation for the surgery, awareness about smoking cessation and other lifestyles, and admission day tips); (2) five educational modules with aspects related to the surgery, which are designed to be used during hospitalization; and (3) eight modules addressing aspects related to postsurgery recovery (eg, skin and wound care, healthy eating, rehabilitation, physical activity). The educational content is personalized based on the patient profile, so contents are related to the specific surgery of the patient and adapted to the patient’s health care provider clinical workflow.

The mobile app also includes *mental well-being activities* designed for stress relief based on mindfulness principles and cognitive behavioral therapy (see Multimedia Appendix 1 for a screenshot). These include a combination of multimedia content, such as nature and music mindfulness exercises, breathing relaxation training, and meditation. When a mobile app detects high levels of reported stress, mental well-being exercises are recommended to users.

The mobile app also addresses the importance of *patient monitoring* of health status and lifestyle to provide personalized care and tailored content. For this reason, the mobile app provides both subjective (eg, patient-reported outcomes based on psychometrics) and objective monitoring using a smartwatch (Withings Pulse HR, Withings France SA) to monitor biometric variables of health outcomes and relevant behaviors (eg, physical activity; refer to Multimedia Appendix 2 for a screenshot). HCPs can monitor these patient parameters via a web application (see the next subsection). In the context of supporting patients during hospitalization, the solution relies on automatic patient tracking using an IoT-based indoor location. Patient monitoring includes multiple questionnaires enabling pain and stress self-assessment as well as wound healing progress. The questionnaires already available include the visual analogue scale (VAS) for stress, VAS for pain, and Bluebelle Wound Healing Questionnaire [34]. Through this module, patients can report potential complications while monitoring the wound healing and recovery time after discharge.

As part of the integration with the existing clinical workflow, the mobile solution provides a *communication module* where patients can chat with their health care team through an asynchronous chat.

One of the key elements of a mobile solution is *personalization*, which maximizes patient engagement. A core element of the personalization strategy is the use of the Adhera HRS, which provides personalized messages to patients based on behavioral change models such as the I-Change model [26]. These messages are tips and pieces of advice about how to adopt healthy behaviors such as quitting smoking before and after surgery to improve recovery length. Patients are guided to adhere to healthier habits and reduce their stress and anxiety levels before and after surgery.

The motivational messages of the digital program have been designed by expert psychologists, clinicians, and researchers in the field of behavioral change models for stress and anxiety relief (refer to Multimedia Appendix 3 for a screenshot). The messages were based on specific motivational and behavioral change factors represented by attitudes. These include factors such as social influence and support in reducing stress and anxiety symptoms, self-efficacy (patients’ perception of their ability to manage stress and anxiety situations), personal skills for self-regulation (in stress and anxiety events), and action planning (actions to perform the desired behaviors and coping or maintaining planning). These factors are encountered in the 3 phases of the behavioral change process: enhancing awareness, increasing motivation, and leading to intentional behavioral change. According to this theoretical framework, the message meta-features were designed first, for each phase and factor, ensuring at least one message for each meta-feature and for each combination of meta-features. Second, motivational messages have been defined also considering sex and age differences, as well as the type of surgery and patient. To provide motivational messages, we relied on the I-Change model, which provides guidance on personalizing motivational messages according to individual patient profiles. These recommendations are sent with the frequency preferred by users and when the AI-based solution detects that a subjective or physiological parameter has increased or decreased (eg, reported high stress level and sensor-derived physical activity), these parameters are only used for personalization of the AI-based recommendations and not for assessing the stress levels. In this case, a stress-relief technique is recommended to the patient depending on the nature of the source of stress. The HRS relies on a profiler module that gathers relevant information from patients about their desired level of detail with regard to surgery information (eg, general or graphical), preferences for techniques to manage stress (music preferences, virtual places, etc), and other relevant information to create personalized interventions (education level, age, medical condition, etc). The stored information is used to adapt the educational and motivational content to individual needs and preferences that the user can change or modify at any time.

The data from the patient profile (type of surgery, preferences, specific requirements, etc) are combined with lifestyle monitored data and implicit and explicit user interaction patterns with the content. This allows the updating of patient information and helps to tailor the best stress management strategy. Furthermore, a real-time location system based on 4 Bluetooth low-energy beacons installed at the hospital entrance, admission desk, ward, and surgery area provides the mobile app with the patient location, so context-aware content is recommended to the patient based on that location (eg, how to navigate the hospital and preparation after admission). IoT-based functionality is not required at home.

### The Digital Health Program: VR Component

#### Overview

**Figure 3:**
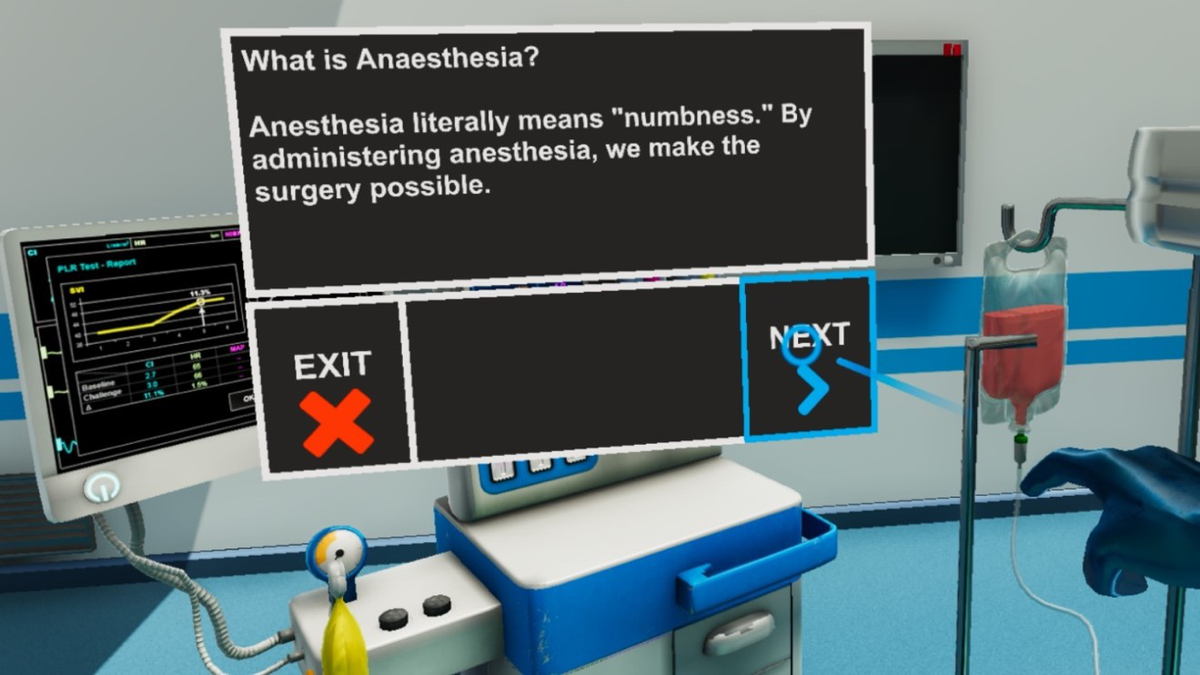
A screenshot of the digital health virtual reality component displaying anesthesia information inside the “OR” environment.

The digital health program used a VR component as an additional channel for delivering patient education, perioperative stress management, and pain relief. The information was presented to the patient via step-by-step guidelines and gamified scenarios, representing learning instructions and procedures to prepare the patient for the actual surgical procedure and the period that will follow the surgery. This content is presented in a gamified way, delivering body and mind exercises, instructions for postsurgery diet, and a postsurgery exercise program. VR implementation represents a novel approach for delivering educational content and knowledge about perioperative care procedures while empowering patients to combat stress. It provides (1) an immersive, gamified educational VR preoperative simulation of the proposed medical procedure to familiarize the patients with each step of the patient journey (Figure 3) and (2) a mindfulness and wellness VR layer for coping with the postoperative effects. This reinforces and complements the interventions delivered by the mobile app.

The CARINAE VR is powered by the Unity-based 3D engine provided by the ORAMA VR M.A.G.E.S. platform [35]. The CARINAE VR component guides users with a virtual companion, who is a friendly robot that addresses users in their native language using both audio and textual feedback. To encourage adoption by health care institutes, the CARINAE VR component is integrated into the Pico G2 4 K headset, which provides a cheaper, easier-to-use, yet high-quality alternative available in the VR headset market.

Users are transported to a serene and calm location (CARINAE Mansion), inviting them to explore and interact with their content. VR applications consist of interactive means in IVR environments. Such activities are complemented by features designed to distract the patient with short, fun exercises and activities aimed at occupying the patient’s focus, reducing stress (in case of an upcoming operation), and distracting them from pain (after surgery). The CARINAE VR component features the following contents:

- The patient enters the *virtual environment*, the CARINAE mansion, located on a remote island during a sunny and relaxing day. When the application was launched, the medical robot introduced basic controls and interacted with the virtual environment. The user becomes familiar with VR controllers and how to access the features of the mobile component. A *multimedia screen* allows the selection of the desired playlist or video organized by genre.
- The patients’ *recommended meals* for each day along with their nutritional facts are provided on the interface table (refer to Multimedia Appendix 4 for a screenshot). The board included the recommended perioperative physical exercises for the patient. Exercises can be selected, and a pop-up window containing additional information and recommendations can be opened for the instructions. Outside the CARINAE mansion, there is also a yoga mat in which the user can perform breathing exercises following animated instructions.
- Maps for in-hospital education and familiarization:
  An *interactive hospital map* guides patients to the hospital. The patient can click the “up” and “down” buttons to see the different floors of the hospital. At the bottom side of the panel, there are selected areas of the hospital that the patient visits during hospitalization, for example, cardiology, surgery room, etc. By clicking on these buttons, the map automatically focuses on and shows the selected position in the hospital.
  The educational module included a *virtual visit to the operating room*. Users can access the “Operation preview” button located on top of the surgical bed to be teleported to the virtual operating room, where a number of information points pose contents related to their upcoming surgery. These information points also include material regarding the risks and possible complications of the surgery, as well as the anesthesia machine, by addressing the most commonly asked questions about the anesthesia process.
- Stress relief VR applications: The VR environment encompasses various *mini games and interaction points*. In particular, a remote-controlled toy robot allows patients to navigate around the room and playfully interface with multiple types of objects in the scene. Entertainment with physical simulations through virtual characters makes the function feel more natural.
  Mini games: A mini game called “whack-a-mole” is available with the VR tool. The game consists of taking a wooden mallet and hitting “animatronic” rodents (moles) that protrude out of holes carved on the playing surface and described in the scientific literature as a beneficial tool to improve essential cognitive skills. Another game, known as stone balancing, allows users to balance, or “stack” stones on top of one another to produce various “sculptures.” This activity is often used in mindfulness-based stress reduction, as it improves patients’ moment awareness.
  Traveling Exercise: A powerful *visualization* (guided imagery) technique integrated into the VR module engages users to bring their imagination into play to mentally “travel” to a positive, peaceful, and calming setting. Guided imagery has been scientifically linked to effective postoperative pain management.
  Whiteboard: This VR application allows users to grab pens of different colors and draw them. Coloring has been suggested to have many mental health benefits, one of which is stress relief. It enables users to focus on a task or moment while stimulating creativity and logic.

#### The Digital Health Program: Clinical Web Application

The web application targets health care providers and provides them with the necessary tools to enroll patients into the digital health program, as well as a tool for following up and communicating with patients. The web application features content about the profile of each patient and their preferences, such as the level of detail that they are interested in about their upcoming surgery, stress management preferences such as music and virtual places, information about their medical history and demographics to facilitate the personalization of the stress-relief content, as well as a dashboard for physicians to facilitate the visualization of patient-reported input over time, such as anxiety and pain levels.

This web application is mainly used to enable HCPs to manage, monitor, and follow patients enrolled in digital health programs. Whenever available, it connects to the electronic health record of the client to automatically retrieve useful information of the users (credentials) and patients enrolled (eg, demographics, medical history, clinical reports, and laboratory tests) as well as to provide relevant information about patients undergoing surgery such as their stress level. The web application includes the following modules.

#### Profiler for Personalization Module

The profiler module gathers relevant information from patients about (1) the level of information they would like to receive regarding their surgery (general, more detailed, and graphical); (2) how they would prefer to manage stress (music preferences, virtual places, etc); and (3) other relevant information to create personalized intervention (education level, age, medical condition, etc). The information stored is adapted to individual needs and preferences that the user can change or modify at any time. All necessary actions were taken to ensure equality of use and avoid age and gender discrimination. Data from the patient profile (type or surgery, preferences, specific requirements, etc) are combined with lifestyle monitored data and implicit and explicit feedback of patients and caregivers when using the mobile app to update patient information and tailor the best stress management coach strategy in accordance with physicians’ recommendations.

#### Communication Module

This module enables communication with the mobile app users. It is based on asynchronous chat that can be used to exchange messages with health providers and contact them in case of an emergency. In addition, predefined messages can be configured to be sent to users at specific time points of the patient journey (ie, the day before the surgery, remind clinical appointments, etc).

#### Lifestyle Monitoring Module

This module enables the longitudinal visualization of the biometric information gathered through the mobile app, including physical activity and other information helpful in understanding patient behavior along the patient journey and monitoring his or her progress between clinical visits.

#### Patient Tracking Module

**Figure 4:**
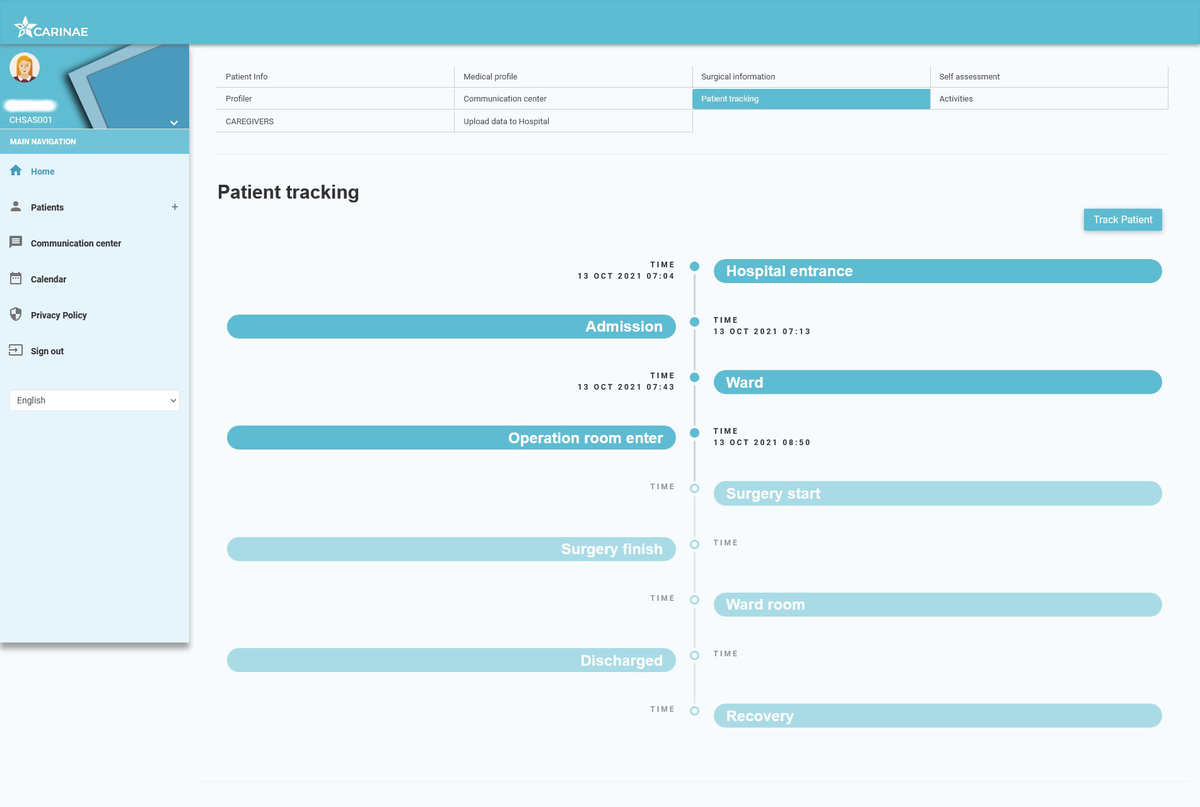
A screenshot of the Digital Health Program web application.

The patient tracking module helps HCPs track patient location and elapsed times between specific steps of the patient journey from admission to hospital discharge. Patients are automatically tracked using BLE beacons strategically installed in the hospital entrance, admission, surgery ward, and presurgery room. From that point onward, HCPs manually update patient tracking, which also triggers notifications to the caregiver’s mobile app, helping them to monitor their relative along the surgical journey (Figure 4).

#### Self-assessment Module

The self-assessment module facilitates visual monitoring of psychometric information through validated patient-reported outcome measures and questionnaires by HCPs. This information includes the VAS for stress and pain and the Bluebelle Wound Healing Questionnaire, which includes pictures of the wound taken with the mobile app. The sampling frequency of these questionnaires was preset according to the advice of psychologists and surgeons.

## Methods

### Overview

The Adhera CARINAE Digital Health Program will be tested in a multicenter trial, including 5 clinical settings across 3 European countries, and will use a stratified randomized controlled design including 30 intervention participants (6 per clinical site) and 30 control group participants (6 per clinical site; Multimedia Appendix 5). This study aimed to obtain objective answers to the following 2 research questions:

1. Is the designed protocol feasible for conducting a future definitive randomized controlled trial? Accordingly, to what extent does the program affect patients’ and caregivers’ stress, anxiety, and pain levels, and secondarily, well-being and overall quality of life, compared with patients receiving the standard of care only?
2. What is the overall usability of the digital health solution based on the experience of patients, caregivers, and HCP?

### Study Setting

After approval from the ethics board, the study will be conducted at the 5 departments of the following European hospitals:

1. Maastricht University Medical Center+ (UMC+; the Netherlands)—Cardiothoracic Surgery Department
2. Hospital Universitario Reina Sofia (SAS; Spain)—Cardiothoracic Surgery Department
3. Instituto di Ricovero e Cura per Anziani (INRCA; Italy)—Urology Department
4. Sant Joan de Déu Hospital (Spain)—Orthopaedics and Traumatology Department for Children
5. Fundació Parc Taulí (Parc Taulí; Spain—Orthopaedics and Traumatology Department for Adults.

### Eligibility Criteria

#### Inclusion Criteria

The study included participants aged 8 to 65 years who underwent one of the following surgeries: cardiopulmonary bypass surgery (Maastricht UMC+); coronary artery bypass surgery (Maastricht UMC+); cardiac valve replacement (SAS, Maastricht UMC+); prostate, kidney, or bladder cancer surgery (INRCA); hip or knee replacement (HSJD; Parc Taulí); maxillofacial surgery (HSJD); orthognathic surgery (HSJD); or scoliosis (HSJD). Furthermore, adult study participants will have to have an Android smartphone and demonstrate basic digital literacy (eg, know how to communicate through instant messaging apps or similar). For children, their caregivers should have an Android smartphone and demonstrate basic digital literacy.

#### Exclusion Criteria

Potential participants who are unable to provide informed consent; communicate in the native language; demonstrate basic digital literacy; present symptoms of dementia; are allergic to dedicated wearable materials, such as steel and silicone; are pregnant; and are enrolled in another clinical trial will be excluded.

### Interventions

#### Overview

**Figure 5:**
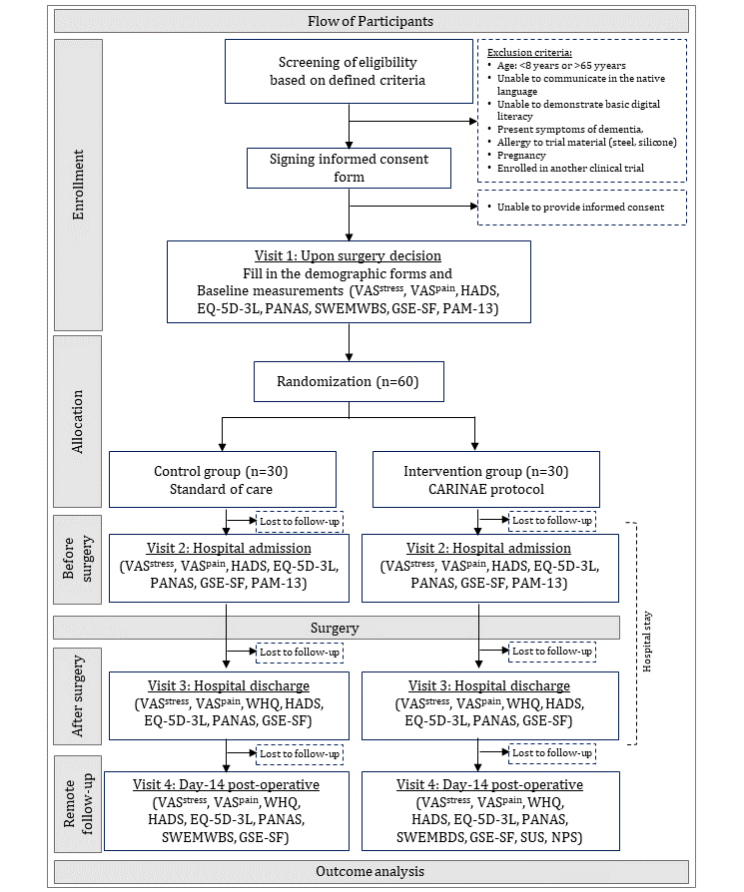
Flow of participants. GSE-SF: General Self-Efficacy Short Form; HADS: Hospital Anxiety and Depression Scale; NPS: Net Promoter Score; PAM-13: Patient Activation Measure short form; PANAS: Positive and Negative Affect Schedule; SUS: System Usability Scale; SWEMWBS: Short Warwick Edinburgh Mental Well-Being Scale; VAS: visual analogue scale.

Eligible participants will be randomly assigned to the experimental group or the control group following block randomization of size 4 using the Sealed Envelope web-based tool [36]. The digital health solution includes 3 distinct components—that is, the mobile app, a VR component, and a clinical web application. The first and second components will be used by the participants in the experimental group, whereas the third will be used only by HCPs for those patients. Both the control and the experimental groups underwent the same visits. The trial was organized as shown in Figure 5.

#### Control Group

The control group will not use the digital solution and will receive standard care consisting of visits to the health care provider. Patients in the control group were provided with instructions on diet and healthy lifestyle habits; however, in current health care settings, it is not very common to provide patients with stress and anxiety perioperative relief support. Assessments of the control group will be performed during the following visits:

1. The initial visit on which the HCP communicates to the patient the need for surgery (2-4 weeks before surgery);
2. Hospital admission (1-3 days before surgery);
3. Hospital discharge (approximately 1 week after surgery);
4. Remote follow-up 14 days after the surgery.

Following each visit, several questionnaires will be administered to patients with a duration ranging from 15 to 45 minutes according to the visit (Table 1).

**Table 1:** Key parameters and measurements: baseline is 2-4 weeks before surgery, (hospital) admission is 1-3 days before surgery, (hospital) discharge is 1 week after surgery, and postoperative is 2 weeks after surgery.

Variables		Participants	Measurements	Baseline	Admission	Discharge	Postoperative
**Primary outcomes**							
	Self-reported stress	Patients and caregivers	VAS^a,stress^	Yes	Yes	Yes	Yes
	Self-reported pain	Patients	VAS^pain^	Yes	Yes	Yes	Yes
	Anxiety and depression	Patients	HADS^b^	Yes	Yes	Yes	Yes
**Secondary outcomes**							
	HRQoL^c^	Patients	EQ-5D-3L	Yes	Yes	Yes	No
	Emotional status	Patients	PANAS^d^	Yes	Yes	Yes	Yes
	Mental well-being	Patients and caregivers	SWEMWBS^e^	Yes	No	No	Yes
	Self-efficacy	Patients and caregivers	GSE-SF^f^	Yes	No	No	Yes
	Activation status	Patients	PAM-13^g^	Yes	Yes	No	Yes
**Usability outcomes**							
	CARINAE’s usability	Intervention group patients, HCPs^h^ and caregivers	SUS^i^	No	Yes	Yes	Yes
	Recommendation grade for CARINAE	Intervention group patients, HCPs and caregivers	NPS^j^	No	Yes	Yes	Yes
	General satisfaction with CARINAE	Intervention group patients, HCPs and caregivers	Ad hoc	No	Yes	Yes	Yes
**Covariates**							
	Demographics	Patients	Self-report assessment questionnaire	Yes	No	No	No
	Clinical data	HCPs	HCP report	Yes	Yes	Yes	Yes
	Length of hospital stay	Patients	HCP report	No	Yes	Yes	No

^a^VAS: visual analogue scale.

^b^HADS: Hospital Anxiety and Depression Scale.

^c^HRQoL: health-related quality of life.

^d^PANAS: Positive and Negative Affect Schedule.

^e^SWEMWBS: Short Warwick Edinburgh Mental Well-Being Scale.

^f^GSE-SF: General Self-Efficacy-Short Form.

^g^PAM-13: Patient Activation Measure short form.

^h^HCP: health care professional.

^i^SUS: Stem Usability Scale.

^j^NPS: Net Promoter Score.

#### Experimental (Intervention) Group

The experimental group will receive a digital health solution during the first visit, and training on how to use this tool will be provided. Patients will take the digital health solution home with them and can use it as often as they like. The experimental group, after each 1 of the 4 standard care visits, will complete the same questionnaires as the control group.

### Outcome Variables

#### Research Question 1 (Primary and Secondary Outcome Variables)

Stress, anxiety, and pain represent the primary outcome variables that will be measured using paper-and-pencil questionnaires.

The secondary outcome variables were overall quality of life, emotional status, mental well-being, self-efficacy perception, and patient activation during and after hospital stay.

To assess the primary outcome variables, the following questionnaires will be used after each standard care visit and will be administered by paper and pencil:

- Patients’ and caregivers’ *self-reported stress* was measured using a VAS at enrollment (baseline), at admission for surgery, at hospital discharge, and 2 weeks after surgery. Operationally, a VAS is usually a horizontal line, 100 mm in length, anchored by word descriptors at each end, such as no stress versus highest stress possible [37].
- Patients’ *self-reported pain* was measured using a VAS for pain measurement at enrollment (baseline), at admission for surgery, at hospital discharge, and 2 weeks after the surgery. The VAS for pain is a validated single-item scale that is anchored by “no pain” (score of 0) and “pain as bad as it could be” or “worst imaginable pain” (score of 100). The VAS presents a high internal consistency, as shown by a Cronbach α of 0.9117 [38].
- Patients’*Hospital Anxiety and Depression Scale* (HADS) scores were measured at admission for surgery, at hospital discharge, and 2 weeks after surgery. The HADS consists of 2 dimensions with 7 questions each, one representing the anxiety subscale and the other representing depression, with both psychopathological concepts of anxiety and depression being independent. Each item was rated on a 4-point frequency scale, ranging from 0 to 3. The internal consistency of the HADS, as measured by the Cronbach α coefficient, was found to be .78 for the anxiety subscale and .86 for the depression subscale, indicating satisfactory reliability [39].

To assess the secondary outcome variables, the following questionnaires will be used after each standard care visit and will be administered paper and pencil:

- Patients’ *health-related quality of life* (HRQoL) was measured using the EQ-5D-3L questionnaire at enrollment, admission for surgery, and at clinical discharge. EQ-5D-3L measures HRQoL across 5 dimensions: mobility, self-care, usual activities, pain or discomfort, and anxiety or depression [40]. These dimensions are scored as “no problems,” “moderate problems,” or “severe problems.”
- Patients’ *emotional status* was measured at enrollment (baseline), at admission for surgery, at hospital discharge, and 2 weeks after surgery using the Positive and Negative Affect Schedule (PANAS). The PANAS is represented by 10-items evaluating positive and negative affectivity in terms of descriptive adjectives (eg, active or upset). Responses ranged from 1 (“never”) to 5 (“very much”). Cronbach α indicated excellent internal consistency for both factors (.90 and .91, respectively) [41].
- Patients’ and caregivers’ * mental well-being* was measured at enrollment and 2 weeks after the surgery using the “Short Warwick Edinburgh Mental Well-Being Scale.” This instrument specifically targeted positive mental functioning. The scale is composed of 7-items (eg, “I’ve been feeling useful”) with a 5-point response range from (“none of the time” to “all of the time”). The questionnaire has high internal consistency reliability, with a Cronbach α of .89 [42].
- Patients’ and caregivers’*self-efficacy* measured at enrollment and 2 weeks after surgery using the General Self-Efficacy in Short Form (GSE) questionnaire. Self-efficacy refers to the general perception of one’s capability to handle adversities. The GSE-SF is a 6-item scale wherein several self-descriptive sentences are scored from 1 (“not at all true”) to 4 (“exactly true”). The questionnaire presents high internal consistency reliability with a Cronbach α of .85 [43].
- Patients’ *activation status* was measured using a self-reported questionnaire, the Patient Activation Measure short form (PAM-13), at enrollment, admission for surgery, and 2 weeks after surgery. PAM-13 is an instrument that assesses patient knowledge, skills, and confidence in the management of chronic conditions [44]. Response options ranged from (1) strongly disagree to (4) strongly agree, and an additional “not applicable” option. The questionnaire presents high internal consistency reliability with a Cronbach α of .81.

#### Research Question 2 (Usability Outcome Variables)

The usability of the intervention tools will be monitored during hospital stay and posthospitalization using well-validated and ad hoc metrics. The *System Usability Scale*, *Net Promoter Score*, and an ad hoc *usability questionnaire* will be administered to the intervention group patients and caregivers as well as the HCPs who are involved in the care management of the intervention group [45,46].

The System Usability Scale is a 10-item evaluation based on a 5-point Likert scale measuring the strength and agreement of usability [45].

The ad hoc *usability questionnaire* assessed 4 dimensions: (1) functionalities, (2) design, (3) manual and instruction for use, and (4) general usability on a Likert scale (1-10). Each dimension also contained open fields to which participants responded qualitatively.

Net Promoter Score is used to assess general satisfaction with the question, “How likely are you to recommend this service?” [46]. The response format ranged from –100 to +100. Specific satisfaction was measured using a Likert scale (0-100) on a single question (“Did the user like the CARINAE solution?”).

#### Independent Variable

The independent variable in this study was the type of intervention. This variable is allocated to 2 levels: control and intervention.

#### Confounding Variables

Patient age, sex, surgery type, medication use, length of hospital stay, and health outcomes, such as postoperative complications, reoperation, mortality, and groups in which the patients were involved during the intervention, will be used as confounding variables.

### Participant Timeline

This feasibility trial consisted of an 8-week intervention treatment phase, including 4 visits; the last 2 weeks will be a follow-up phase, and visit 4 will be performed remotely. The total trial data collection period will be 2 months. The measurements were performed as shown in Table 1.

### Sample Size

As this was a feasibility study, a sample size calculation is not required. However, we can estimate the number of participants we will be able to recruit during the data-collection period. In this feasibility study with a statistical power of 0.8, an α of .05, and an effect size of 1.20, the minimum required sample size will be 60 participants (30 in each group). Sample size calculations are estimated using G*Power (version 3.1.9.2).

### Recruitment

#### Participants

Clinical investigators will prescreen the eligibility of the participants that they have available in their pool of subjects proposed for one of the surgeries in the inclusion criteria. Whenever they find a potentially eligible participant, they will invite them to participate in the study, either by phone or during routine consultation, whichever is more convenient. If the patient shows interest in participating in the study, then he or she will be referred to the research coordinator of the study that will facilitate him or her the patient information letter and the informed consent form and will solve any questions and concerns the patient may have. Upon obtaining informed consent, the patients will be considered recruited for the trial.

#### Randomization and Allocation

Participants-patients will be randomized through the prestratified randomization method to achieve balance between the 2 groups (intervention and control groups) according to the type of surgery and baseline characteristics (covariates). Stratified randomization was achieved by generating a separate block for each combination of covariates and participants were assigned to the appropriate block of covariates. The specific covariates in the clinical trial were represented by the type of surgery (3 surgeries) and sex (2 levels: male and female). With these 2 covariates, the possible block combinations totaled 6 (eg, male, cardiac valve replacement). According to this, the participants’ allocation will be blinded through an Excel file spreadsheet (RAND function) using permuted block randomization with a ratio of 1:1 between the intervention and control groups. Allocation concealment will be ensured, as we will not release the randomization code until the patients are recruited for the trial.

### Data Analyses

The analyses will be conducted in Python 3.9.5 through the IDE PyCharm Community Edition 2021.2.1 Software and intention-to-treat principles. Descriptive statistics will be used to characterize the groups during the 4 visits. Furthermore, owing to the small sample size, Spearman correlations will be performed with all the variables to check, first, for potential associations between the variables and, second, differences between the intervention versus control groups. Further potential differences between these groups will be analyzed as follows:

- Stress (VAS), pain (VAS), and mood state (PANAS-short form) will be subjected to a multivariate mixed analysis of variance (split-plot ANOVA) with “time” as a within-subject factor (ie, 4 time points) and “group” as a between-subject factor (intervention vs control).
- Patient activation (PAM-13) and self-efficacy (GSE-SF) will also be submitted to a multivariate mixed analysis of variance with “time” as a within-subject factor (Ie, 3 time points) and “group” as a between-subject factor (intervention vs control).
- Mental well-being (Short Warwick Edinburgh Mental Well-Being Scale) and HRQoL (EQ-5D-3L and EQ-5D-Y) will be analyzed using multivariate mixed analyses of covariance. As measurements of these 2 variables are based on a pre-post design, postmeasurements will be used as the dependent variable and premeasurements as covariates.

Finally, it is of interest to explore the effects as a function of different types of surgery. To this end, 2 different statistical approaches will be used:

- Cluster analyses per surgery type. Data will be split for each of the surgeries, and variables will be analyzed individually using time and group as within and between factors, respectively.
- A different statistical approach from the approach described above will also be used. In this case, analyses are performed within a linear mixed model (LMM) framework. LMM allows for the flexible handling of unbalanced data, outliers, or missing observations without averaging the data of participants. In addition, LMM enables the modeling of data using different fixed and random factors. Accordingly, each of the variables will be submitted to an LMM analysis wherein “time,” “group (intervention vs control)” and their interaction (time × group) will be included as fixed factors. In contrast, “subjects” and “surgery” will be submitted as random factors. This analysis enables the calculation of regression estimation parameters by considering that participants differ in the surgery type. In addition, the participants’ age or sex may also be used as control covariates.

### Ethics and Dissemination

#### Ethics Approval and Consent to Participate

All procedures were approved by the ethics committees of the 5 clinical sites in the 3 countries involved (Italy, Netherlands, and Spain):

1. Maastricht UMC+ (the Netherlands)—Cardiothoracic Surgery Department
2. Hospital Universitario Reina Sofia (SAS; Spain)—Cardiothoracic Surgery Department
3. INRCA (Italy)—Urology Department
4. Sant Joan de Déu Hospital (Spain)—Orthopedics and Traumatology Department for Children
5. Fundació Parc Taulí (Parc Taulí; Spain)—Orthopedics and Traumatology Department for Adults.

Spanish regulation allows only 1 ethics committee to be requested per country, and its approval applies to the national territory. Approval in Spain was requested by the Hospital Reina Sofía (SAS) ethical committee (Stars-car-0320-5113) and has been acknowledged by both Hospital Sant Joan de Déu and Parc Taulí ethical committees.

Signed consent was obtained from all participants in the study. For those who are unable to provide informed consent, the caregiver will approach each potential participant and his or her substitute decision maker to provide information on the study. If these potential participants and their substitute decision makers provide their consent, the substitute decision makers will sign the consent form, and the HCPs will seek the potential participants’ assent. Participants will not receive any incentive to participate in this study.

#### Withdrawal From the Study

The participants and substitute decision makers can request to withdraw from the study at any time, either orally or in writing. The participants will be able to withdraw from the study at any time before the group analysis is calculated. If a participant withdraws, his or her information will not be considered in the analysis. If a participant requests to have his or her data be destroyed, the research team will honor this request by shredding and recycling the paper records and erasing any records stored on a computer hard drive using commercial software applications designed to remove all data from storage devices. However, once all the participants’ data were analyzed, the participant could not withdraw. Participants will be informed of this condition in a consent letter. The deadline date for withdrawal will be determined once all participants’ data have been collected, and data analysis is underway. This will occur during the 6 months of the study.

#### Consent or Assent

Signed consent will be obtained from all participants in the study. For those who are unable to provide informed consent, the caregiver will approach each potential participant and his or her substitute decision maker to provide information on the study. If these potential participants and their substitute decision makers provide their consent, the substitute decision makers will sign the consent form, and the HCPs will seek the potential participants’ assent.

#### Confidentiality

Participants will be assigned an alphanumeric code instead of using their names or other identifiers. Only the study coordinator will have access to the master list, where these codes are linked to the participants’ first names. With the exception of direct conversations with each participant, their names will not be used, only their numbers. Hard copies of the consent forms, questionnaires, and study notes will be stored in a locked filing cabinet in the 5 departments of the 5 hospitals. All of the deidentified electronic study documents will be encrypted and stored on a password-protected computer drive.

#### Access to Data

Participating HCPs will be given access to cleaned data sets. The master list will be stored on a password-protected computer. Only the study coordinator will have access to the master list. The data will be retained for 5 years and will be published in peer-reviewed journals and conferences. The data will not become part of a data repository and will not be involved in the creation of a research database or registry for future research. After 5 years, the data will be destroyed.

#### Quality Assurance and Safety

We will follow the CONSORT (Consolidated Standards of Reporting Trials) guidelines for clinical trial feasibility.

## Results

The recruitment process started in January 2022 and has been completed. The primary impact analysis is currently ongoing. The expected results will be published in early 2023.

## Discussion

### Overview

The primary objective of the proposed study is to assess the feasibility of a digital health support program and its related technologies, that is, wearables, mobile apps, and VR for reducing perioperative stress. The results of this project will inform the development of best practices for patients’ stress relief during the perioperative process.

Several strategies and techniques have been proposed to manage preoperative stress and anxiety, which can be effective in supporting patients to cope with a wide range of stressful health situations. In current health care settings, however, it is not very common to provide patients with stress and anxiety relief support before a surgical procedure. The available VR apps focus only on providing informative content, neglecting the importance of patient empowerment with a more robust educational curriculum. This study will be among the first to evaluate the potential effectiveness of complete IVR technology for reducing perioperative stress.

This protocol describes a complete digital health solution that offers a unique combination of end points and the integration of knowledge derived from several domains, including stress and anxiety management, patient empowerment, communication with medical professionals, adaptation to illness, self-regulation, self-management, and adaptation to medical procedures.

This digital health solution allowed participants to receive constant feedback to improve their appraisal and coping skills in an entertaining and motivating manner. It focuses on patient empowerment through active participation in the process and is dynamically adapted according to operation type, patient preferences, needs, and medical history throughout the preclinical phase, admission, and discharge in a continuous and personalized manner. Simultaneously, it facilitates effective interactions between patients and HCPs through user-friendly and intelligent communication. It uses the spaced learning methodology to help patients understand and learn the diverse aspects of their surgical process, from presurgery requirements to recovery steps with stress management throughout the process and provides multichannel anxiety and stress-relief personalized content.

The authors believe that the digital health solution, combining mobile health and VR technologies with a web application, can provide positive results in reducing perioperative stress and creating effective collaborations between physicians, surgeons and their patients, while supporting them in improving their knowledge in related domains. The Adhera CARINAE Digital Health Program has the potential to improve physical and emotional reactions to stressors, such as surgical operations, increase the levels of calmness, promote a sense of well-being, and empower patients in preoperative conditions. Information provided through the platform advances and enhances health literacy and digital competence and increases the participation of the patient in the decision-making process. Integration with third-party apps can facilitate the exchange of important information between patients and physicians as well as between personal applications and clinical health systems.

### Limitations

Information provided through the digital health solution is not offered in the control group. Moreover, the digital health solution offers personalized information to the intervention group, which was not possible in the control group. This introduces a bias in the measurement results. Furthermore, for pediatric patients, as the intervention was observed and materialized through their caregivers, additional bias was introduced in the measurements.

### Conclusions

This protocol defines the application of a very innovative approach to the management of stress associated with painful and stressful medical procedures, such as surgery. The project depicted in this manuscript proposes an excellent tool that companies and the health care system can use to collaborate on promoting the entry of this valuable technology into the global health care system market.

In conclusion, this study will provide valuable information on the effects of digital health interventions in comparison with the standard of care. A great deal of insights on the comparative effects of the tool on relevant outcomes and the usability of the comprehensive digital health solution by patients, caregivers, and HCPs will be acquired. This will contribute to the evidence planning process, which is crucial for future adoption of digital health programs.

## Appendix

Multimedia Appendix 1 Mental well-being activities.

Multimedia Appendix 2 Mobile health app biometric synchronization.

Multimedia Appendix 3 Personalized motivational messages.

Multimedia Appendix 4 A screenshot of the CARINAE virtual reality app displaying perioperative nutrition information inside the “mansion” environment.

Multimedia Appendix 5 Summarized SPIRIT (Standard Protocol Items: Recommendations for Interventional Trials) table.

## Abbreviations

AI: artificial intelligence
CONSORT: Consolidated Standards of Reporting Trials
HADS: Hospital Anxiety and Depression Scale
HCP: health care professional
HRQoL: health-related quality of life
HRS: health recommender system
INRCA: Instituto di Ricovero e Cura per Anziani
IoT: Internet of Things
IVR: immersive virtual reality
LMM: linear mixed model
PANAS: Positive and Negative Affect Schedule
UMC+: University Medical Center+
VAS: visual analogue scale
VR: virtual reality

## Data Availability

Data sharing is not applicable to this study because no data sets were generated or analyzed during the study protocol generation.
