# Supplementary figures and images for: A Digital Health Intervention for Stress and Anxiety Relief in Perioperative Care: Protocol for a Feasibility Randomized Controlled Trial
Source: JMIR Res Protoc. 2022 Nov 29;11(11):e38536. doi: 10.2196/38536 (PMC9748793; doi:10.2196/38536)

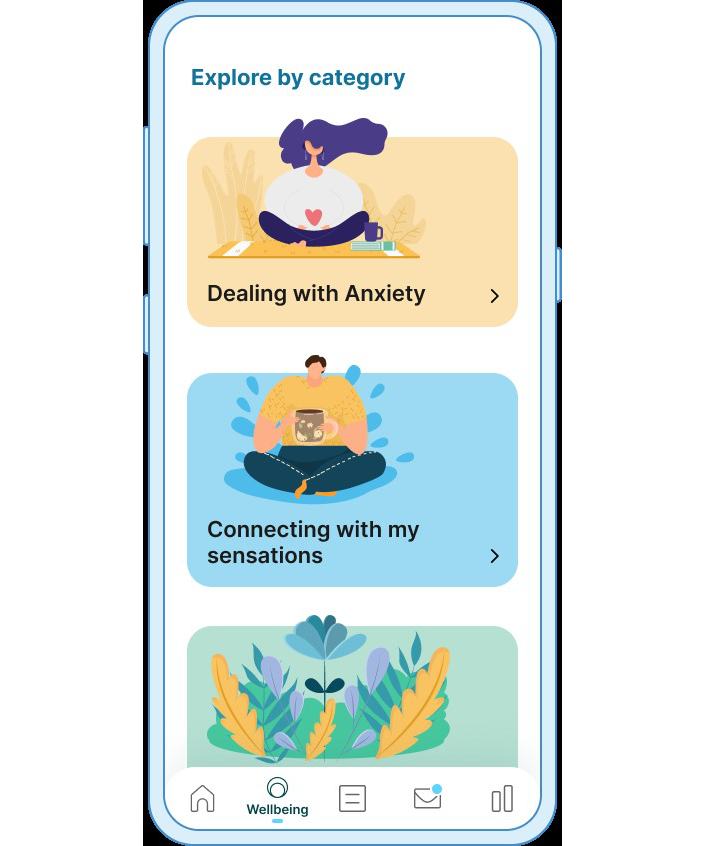

Supplement: Multimedia Appendix 1 [file resprot_v11i11e38536_app1.png]

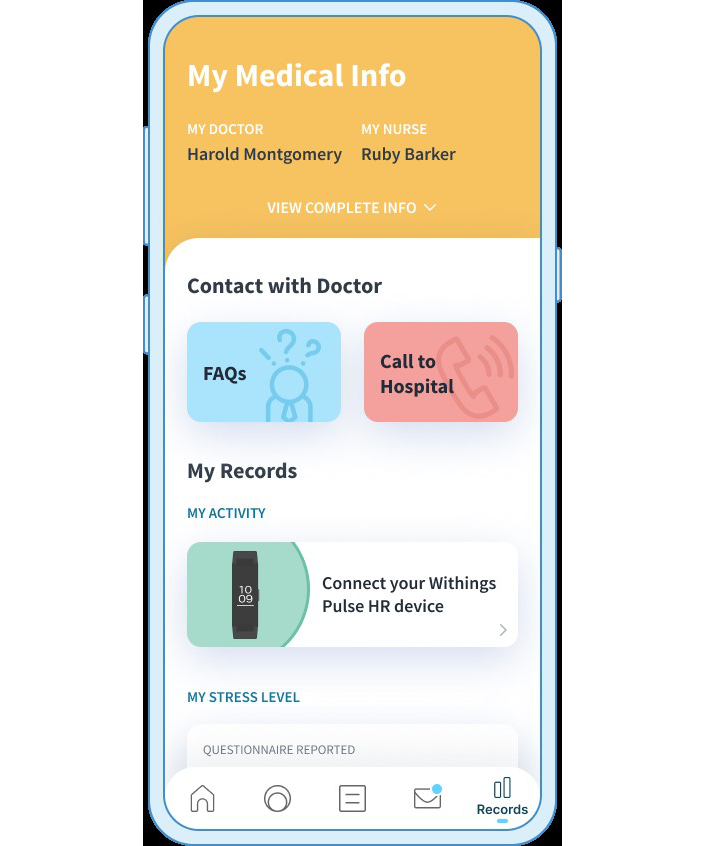

Supplement: Multimedia Appendix 2 [file resprot_v11i11e38536_app2.png]

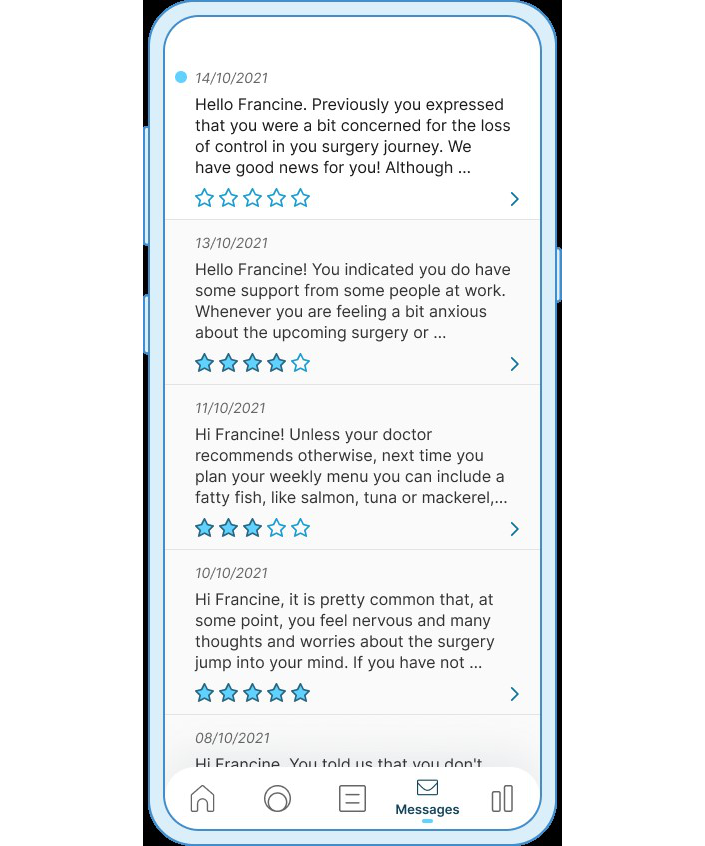

Supplement: Multimedia Appendix 3 [file resprot_v11i11e38536_app3.png]

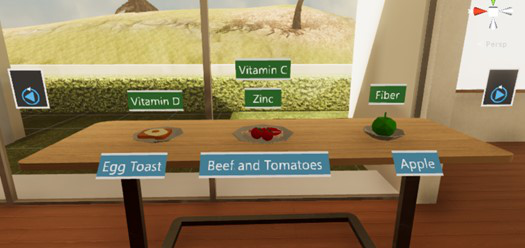

Supplement: Multimedia Appendix 4 [file resprot_v11i11e38536_app4.png]
