# Supplementary material for: A Digital Health Intervention for Stress and Anxiety Relief in Perioperative Care: Protocol for a Feasibility Randomized Controlled Trial
Source: JMIR Res Protoc. 2022 Nov 29;11(11):e38536. doi: 10.2196/38536 (PMC9748793; doi:10.2196/38536)
Supplement: Multimedia Appendix 5 [file resprot_v11i11e38536_app5.doc]

Summarised SPIRIT Table

| Section/item | ItemNo | Description |
| --- | --- | --- |
| **Administrative information** | | |
| Title | 1 | A Digital Health Intervention for Stress and Anxiety Relief in Perioperative Care: Protocol for a Feasibility Trial. |
| Trial registration | 2a | NCT05184725 |
| Protocol version | 3 | 1.1 |
| Funding | 4 | This project has received **funding** from the European Union's Horizon 2020 research and innovation programme under **grant** agreement no. 727585 |
| Introduction |  |  |
| Background and rationale | 6a | Preventing pre-surgical stress can help patients achieve positive outcomes on health and well-being. However, very few patients receive adequate stress relief support prior to a surgical procedure. Provision of education and information about the surgery can be a crucial component of the preoperative experience and is inversely related to levels of preoperative anxiety. However, resource constraints make face-to-face education sessions untenable, given cost considerations and time investment by trained health personnel. Interventions based on mobile health (mHealth) technologies, geared towards increasing familiarity with surgical procedures and hospital environments have been shown to help patients feel informed about possible benefits and risks of available treatment options. mHealth apps and Virtual Reality (VR) can offer patients experience in the perioperative environment that can be helpful in empowering patients and enhancing a more positive experience, while reducing stress. However, available applications focus only on providing informative content, neglecting the importance of patient empowerment with a more robust educational curriculum. |
| Objectives | 7 | Psychological Stress |
| Trial design | 8 | |  |  | | --- | --- | | Study Type  : | Interventional  (Clinical Trial) | | Estimated Enrollment  : | 60 participants | | Allocation: | Randomized | | Intervention Model: | Parallel Assignment | | Intervention Model Description: | Digital Health Intervention. | | Masking: | None (Open Label) | | Primary Purpose: | Treatment | |
| Methods: Participants, interventions, and outcomes | | |
| Study setting | 9 | Hospital |
| Eligibility criteria | 10 | Inclusion and exclusion criteria for participants. If applicable, eligibility criteria for study centres and individuals who will perform the interventions (eg, surgeons, psychotherapists) |
| Interventions | 11a | Intervention group will be exposed to the use of SaMD CARINAE for 2 months approximately, a patientcentred digital health support program. The intervention trial will include a total of 4 visits: 1. Baseline (2-4 weeks before surgery); 2. Hospital admission (1-3 days before surgery); 3. Hospital discharge (1 week after the surgery approx.); 4. Post-operative day 14 (2 weeks after the surgery approx). After each visit and intervention trial with SaMD CARINAE the experimental group answer the same questionnaires of the control group, above mentioned Participants allocated to the intervention group will also be asked to complete questionnaires about usability, satisfaction and subjective experience. |
| Outcomes | 12 | Primary Outcome: Visual Analog Scale for Stress |
| Participant timeline | 13 | See Figure in Manuscript |
| Sample size | 14 | 60 |
| Recruitment | 15 | Recruitment at clinical setting inviting participants from elective surgery |
| **Methods: Assignment of interventions (for controlled trials)** | | |
| Allocation: |  |  |
| Sequence generation | 16a | Method of generating the allocation sequence (eg, computer-generated random numbers), and list of any factors for stratification. To reduce predictability of a random sequence, details of any planned restriction (eg, blocking) should be provided in a separate document that is unavailable to those who enrol participants or assign interventions |
| Allocation concealment mechanism | 16b | Mechanism of implementing the allocation sequence (eg, central telephone; sequentially numbered, opaque, sealed envelopes), describing any steps to conceal the sequence until interventions are assigned |
| Implementation | 16c | Who will generate the allocation sequence, who will enrol participants, and who will assign participants to interventions |
| Ethics and dissemination | | |
| Research ethics approval | 24 | Multicentric ethical approval |
| Consent or assent | 26a | Inform consent from patients and caregivers when applicable. |
| Confidentiality | 27 | According to ISO 27001 guidelines and local regulation |
| Declaration of interests | 28 | This is a study sponsored by a commercial entity |
| Access to data | 29 | Upon reasonable request considering privacy agreement and ethical approvals |
| Ancillary and post-trial care | 30 | Provisions, if any, for ancillary and post-trial care, and for compensation to those who suffer harm from trial participation |
| Dissemination policy | 31a | Plans for publication of results in scientific journals. |
|  | 31c | Upon reasonable request |
